# Preoperative prediction of extrathyroidal extension: radiomics signature based on multimodal ultrasound to papillary thyroid carcinoma

**DOI:** 10.1186/s12880-023-01049-8

**Published:** 2023-07-20

**Authors:** Fang Wan, Wen He, Wei Zhang, Yukang Zhang, Hongxia Zhang, Yang Guang

**Affiliations:** Department of Ultrasound, Beijing Tiantan Hospital, Capital Medical University, No. 119 West Road of South 4th Ring Road, Fengtai District, 100160 Beijing, China

**Keywords:** Multimodal ultrasound, Extrathyroidal extension, Radiomics signature

## Abstract

**Background:**

There is a recognized need for additional approaches to improve the accuracy of extrathyroidal extension (ETE) diagnosis in papillary thyroid carcinoma (PTC) before surgery. Up to now, multimodal ultrasound has been widely applied in disease diagnosis. We investigated the value of radiomic features extracted from multimodal ultrasound in the preoperative prediction of ETE.

**Methods:**

We retrospectively pathologically confirmed PTC lesions in 235 patients from January 2019 to April 2022 in our hospital, including 45 ETE lesions and 205 non-ETE lesions. MaZda software was employed to obtain radiomics parameters in multimodal sonography. The most valuable radiomics features were selected by the Fisher coefficient, mutual information, probability of classification error and average correlation coefficient methods (F + MI + PA) in combination with the least absolute shrinkage and selection operator (LASSO) method. Finally, the multimodal model was developed by incorporating the clinical records and radiomics features through fivefold cross-validation with a linear support vector machine algorithm. The predictive performance was evaluated by sensitivity, specificity, accuracy, F1 scores and the area under the receiver operating characteristic curve (AUC) in the training and test sets.

**Results:**

A total of 5972 radiomics features were extracted from multimodal sonography, and the 13 most valuable radiomics features were selected from the training set using the F + MI + PA method combined with LASSO regression. The multimodal prediction model yielded AUCs of 0.911 (95% CI 0.866–0.957) and 0.716 (95% CI 0.522–0.910) in the cross-validation and test sets, respectively. The multimodal model and radiomics model showed good discrimination between ETE and non-ETE lesions.

**Conclusion:**

Radiomics features based on multimodal ultrasonography could play a promising role in detecting ETE before surgery.

**Supplementary Information:**

The online version contains supplementary material available at 10.1186/s12880-023-01049-8.

## Introduction

Extrathyroidal extension (ETE) reflects the spread of a primary thyroid tumour beyond the thyroid gland capsule. It appears in 5–45% of papillary thyroid carcinomas (PTCs) according to previous research [1]. Depending on the extent of invasion, ETE is subclassified as gross ETE (gETE) and minimal ETE (mETE). gETE is regarded as a macroscopic event that was initially suspected or identified by intraoperative, radiologic, or clinical examination and is associated with disease recurrence and survival. mETE is defined as microscopically detected invasion into perithyroidal soft tissue [2]. However, the importance of mETE remains a much-debated topic over the years. However, the 8th American Joint Committee on Cancer (AJCC) no longer includes mETE as the protocol to define T3 for tumour staging of PTC [3]. Some studies have shown that mETE in patients with PTC presents aggressive biological behaviour and is closely related to the risk of recurrence and metastasis [4, 5].

Ultrasound (US) is the first choice for the diagnosis of thyroid cancer and suspicious cervical lymphadenopathy. When physicians evaluate the risk of malignancy through sonographic patterns, evidence of ETE is also the point of examination according to the 2015 American Thyroid Association (ATA) Management Guidelines [6]. Previous work has only focused on the visible characteristics of US while ignoring the limitations of human visual resolution. It has been reported that US estimation of minimal ETE is less sensitive (30.1%) [7]. Thus, there is a recognized need for additional approaches to improve the accuracy of ETE diagnosis on US examinations before surgery. Radiomics allows the rapid quantitative extraction of countless high-throughput features from digital images and is widely used to solve medical problems, such as predicting malignant disease and lymph metastasis [8]. Recent research on US estimation of ETE has been carried out with radiomics based on B-mode images. This approach has enabled more precise prediction of ETE [9].

Extensive research has shown that multimodal US, including B-mode US (BMUS), colour Doppler flow imaging (CDFI), shear-wave elastography (SWE), superb microvascular imaging (SMI) and contrast-enhanced US (CEUS), has been frequently applied to differentiate benign and malignant lesions in the thyroid, breast and liver [10–12]. Hard malignancy as measured with elastographic results was significantly associated with pathological extrathyroidal extension [13]. Quantitative CEUS analysis showed that the time from peak to one-half has good diagnostic value in detecting ETE [14]. To date, far too little attention has been given to predicting ETE by multimodal US radiomics. In this research, we aim to develop and validate a state-of-the-art radiomics model based on clinical records and multimodal US for predicting ETE in PTC patients. Our findings should contribute to the field of noninvasive assessment of ETE and clinical decisions about PTC.

## Methods

The study was approved by the Ethical Committee of the Beijing Tiantan Hospital of Capital Medical University and complied with the Declaration of Helsinki. All patients signed informed consent before the CEUS examination.

### Patients

This retrospective trial assessed consecutive individuals with thyroid nodules first diagnosed from January 2019 to April 2022 at Beijing Tiantan Hospital. All patients were examined by multimodal US and subsequently administered thyroid surgery, subtotal or total thyroidectomy, within a month following the US examination.

The inclusion criteria were as follows: (1) age >= 18 years; (2) primary PTC confirmed after surgery; and (3) multimodal US performed one month before surgery. The exclusion criteria were as follows: (1) preoperative anticancer therapy (radiotherapy, chemotherapy, etc.); (2) no association of pathological results with US imaging findings; (3) poor US quality; and (4) insufficient pathological samples for the assessment of ETE.

Based on a previous study [9], with an ETE prevalence of 20%, a sensitivity of 70%, and a specificity of 80%, the necessary sample size was calculated to be 47 ETE cases and 188 non-ETE cases to detect such a difference at a two-sided α level of 0.05 with 80% power. We used all available data to maximize the power and generalizability of our results.

### Clinical and laboratory information

The following clinical and laboratory variables were retrospectively considered: age, sex, tumour location and thyroid function laboratory test results. All data were recorded before the operation. Thyroid function tests, including thyroglobulin (Tg), thyroid peroxidase antibody (TPOAb), and thyroglobulin antibody (TgAb), were performed in our hospital within one month before surgery [15]. The laboratory results were classified as low/normal/high based on comparison with the normal range, and missing data were imputed using the median value.

### Ultrasound examination

Two sonologists with more than 5 years of experience in thyroid US performed examinations in this study. For each tumour, BMUS, SWE, SMI and CEUS images were acquired on an Aplio 900 or Aplio 500 US system (Toshiba, Tokyo, Japan) with a linear array transducer i18LX5 probe. BMUS scans were first performed to find the optimal scanning plane. Then, CDFI, SMI, SWE and CEUS examinations were performed. For CEUS imaging, 5 mL of the sulphur hexafluoride microbubble contrast agent SonoVue (Bracco SpA, Milan, Italy) was injected into the median cubital vein of patients. Data acquisition was started after injection and lasted for at least 120 s. After that, sonologists performed a neck ultrasound to report whether suspicious cervical lymph nodes existed. Images from BMUS, CDFI, SMI, SWE and CEUS were exported in BMP format for further analysis. The frame at the peak intensity in the CEUS video was used to analyse the enhancement pattern.

### Image interpretation and analysis

**Fig. 1:**
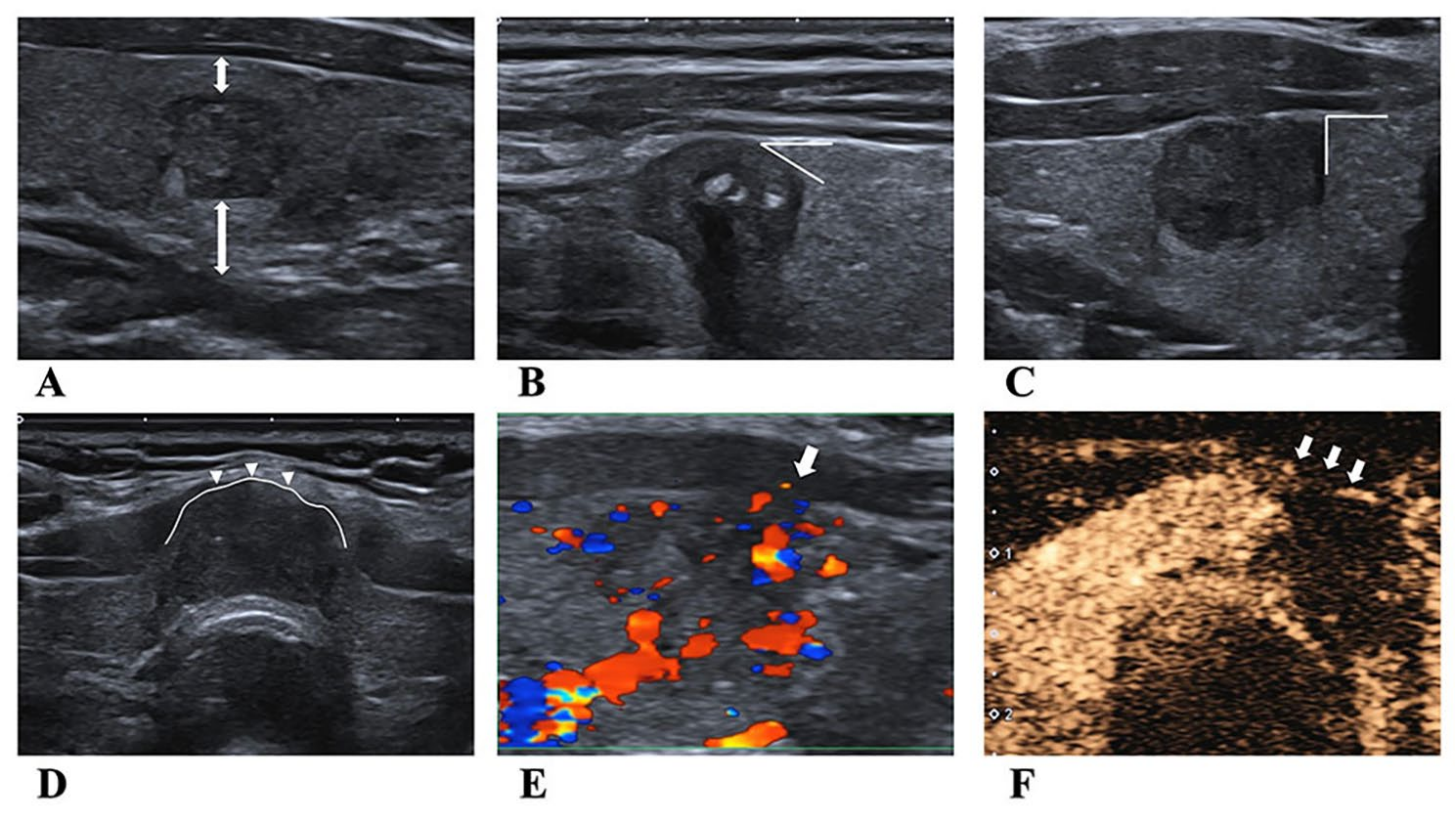
Ultrasonography for diagnosis of extrathyroidal extension (**A**) The lesion is not in contact with the capsule. According to the classification criteria, the contact angle with the thyroid capsule is 0°. (**B**) The lesion formed an acute angle with the thyroid capsule. (**C**) The lesion formed a straight angle with the thyroid capsule. (**D**) Thyroid capsule deformation for the bulging nodule. (**E**) The arrow indicates that the CDFI vascularity of suspicious extrathyroidal extension nodules extended beyond the thyroid capsule. (**F**) The arrows pointing at contrast-enhanced ultrasound imaging showed discontinuous enhancement of the anterior thyroid capsule

The multimodal US characteristics of nodules were evaluated during real-time and multiview scanning before surgery. According to Lamartina L et al. [7] and Zhang Y et al. [16], the BMUS signs of ETE diagnosis were classified as follows: the angle of contact with the thyroid capsule (absent capsule contact, 0; acute, < 90°; straight or obtuse, = or > 90°), the degree of contact (< 25%, 25–50% or > 50% circumference of thyroid nodule contact with the capsule), bulging (nodule that deforms thyroid contour and bulges out), and capsule echogenic loss. The CDFI and SMI characteristics of suspicious ETE were vascularity extending beyond the capsule. The CEUS characteristics of suspicious ETE were classified as follows: (1) discontinuous capsular enhancement (the enhancement of the anterior and/or posterior hyperechoic thyroid capsular was discontinued) and (2) enhancement extending beyond the capsule (enhancement extended out of the capsule). The ultrasonographic representation for the diagnosis of extrathyroidal extension on US is shown in Fig. 1. The 2015 ATA guidelines were used to guide reporting cervical lymph node metastasis on US (US-LNM) [6].

### Radiomics feature extraction

**Fig. 2:**
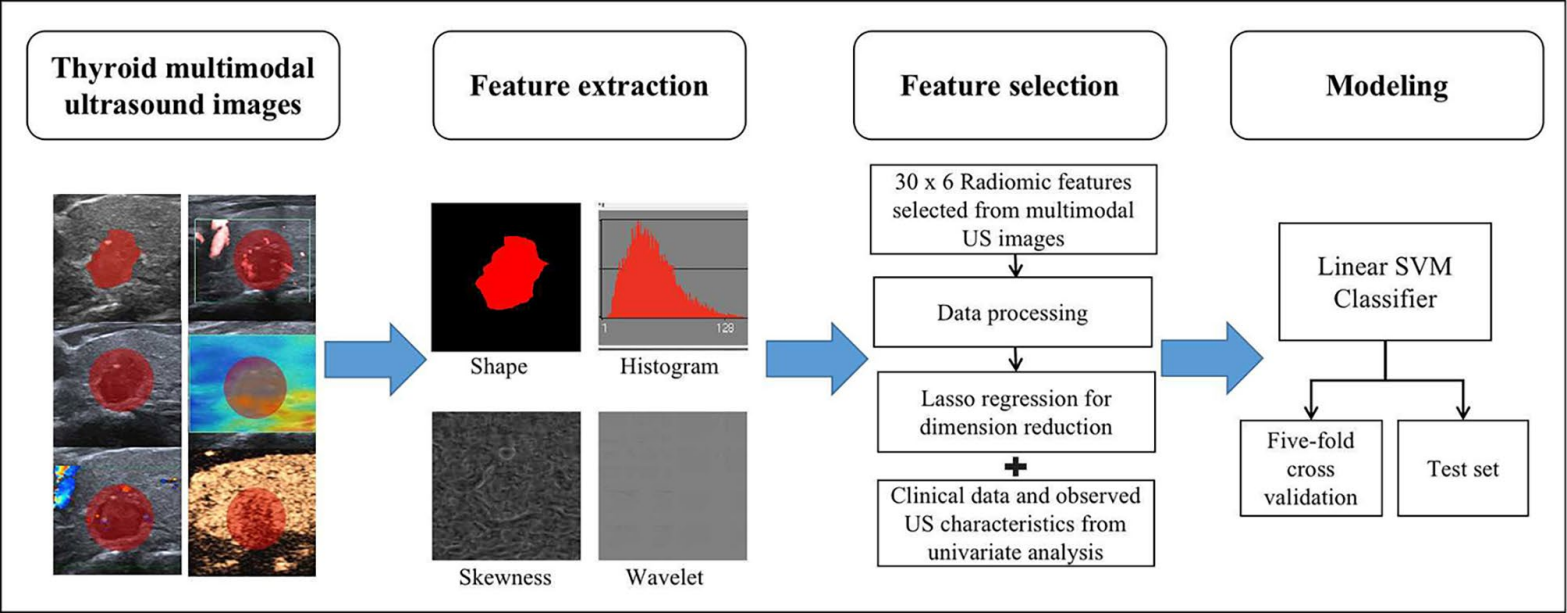
The overall study process The most representative image of each tumour on the thyroid multimodal ultrasound image was selected. Radiomics features, including shape, histogram, absolute gradient, grey-level co-occurrence matrix, run length matrix, autoregressive model, and wavelet transform, were extracted. Radiomics features were generated using the Fisher coefficient, mutual information, probability of classification error and average correlation coefficient methods (F + MI + PA) and LASSO. These selected features were used to train the linear SVM in fivefold cross-validation and test in an test set. Univariate analysis was performed to determine the association between the clinical variables and ETE. Another SVM classifier was built using radiomics features plus clinical variables and observed ultrasound characteristics. SVM: support vector machine

MaZda software (version 4.6, the Institute of Electronics, Technical University of Lodz, http://www.eletel.p.lodz.pl/programy/mazda/) was used to extracted radiomics features from each lesion [17]. We applied image normalization to µ ± 3δ (µ: mean grey-level value, δ: standard deviation) to decrease intensity bias from different images [18, 19]. Radiomics features were automatically extracted from the region of interest (ROI) on the US image of the largest cross-section with MaZda software, including shape features and six common texture feature groups (histogram, absolute gradient, grey-level co-occurrence matrix, run length matrix, autoregressive model, and wavelet transform). Shape features were extracted from the actual ROI, which was manually delineated along the border of each tumour on the BMUS images. Considering that the periphery of the tumour also contained helpful information, other texture features were extracted from the disk structure dilated from the original segmented ROIs (the dilated ROI had a diameter approximately 10% larger than the contour of the lesion) [20]. The reference point to create all the dilated ROIs was the gravity centre of the original ROI [21]. Because the CDFI and SWE images are three channels (RGB), we performed the colour analysis module function to extract features from each channel separately. The overall study process is shown in Fig. 2.

### Intraobserver and interobserver agreement

Given the importance of radiological signature reproducibility, 20 samples were randomly selected to measure the interobserver agreement. The multimodal radiomics features of these samples were extracted by two independent radiologists (Guang Yang and Yukang Zhang) who had at least five years of experience performing US examinations and were blinded to patients’ clinicopathological records. The first radiologist delineated the ROIs of these samples again after two weeks. Then, the delineation of the remaining images was completed by the first radiologist.

### Dimensionality reduction and radiomics feature selection

To avoid the problem of dimensionality and reduce the bias in feature modelling, 30 optimal features were selected from shape features for BMUS, and 150 texture features were selected from texture features for BMUS, CDFI, SWE, SMI, and CEUS based on the Fisher coefficient, mutual information, probability of classification error and average correlation coefficient methods (F + MI + PA). After the normalization of radiomics data, least absolute shrinkage and selection operator (LASSO) regression was performed for dimensionality reduction. The selected features were used to further develop prediction models.

### Development and validation of ETE-predictive models

We randomly selected 74% of the samples as the training set, and the remaining 26% constituted the test set. The models were trained separately in the training dataset and were likewise validated independently in the testing dataset. Candidate variables included demographics, laboratory tests, selected radiomics features and observed multimodal characteristics that were risk factors for ETE (P < 0.05). To uncover the incremental value of the radiomics signatures to the risk for ETE estimation, both radiomics models and clinical models were developed. The multimodal model incorporated radiomics features and clinical risk factors based on a support vector machine with a linear kernel (linear SVM). A fivefold cross-validation protocol with randomly split training data was used to adjust the optimal weight parameters, and the accuracy for each fold was estimated to prevent overfitting. The performance of the models was evaluated and compared with respect to sensitivity, specificity, accuracy, and F1 scores. Receiver operating curves (ROCs) and precision-recall curves (PR curves) were plotted and quantified with the area under the curve (AUC) to evaluate the discriminatory performance between different models in the cross-validation and test cohorts.

### Pathology

All patients underwent hemithyroidectomy or total thyroidectomy. Tissue slices from all patients were independently reviewed by our institutional pathologist (with more than 15 years of experience) to confirm whether the tumour had ETE, which was defined as a tumour extending through the capsule only. mETE was defined as microscopic invasion of adjacent connective tissue, and gETE was defined as gross invasion involving perithyroidal strap muscles or beyond subcutaneous soft tissues, larynx, trachea, oesophagus, or recurrent laryngeal nerve [22].

### Statistical analysis

Statistical analysis was conducted with SPSS 26.0 (SPSS, Chicago, United States) and MedCalc version 20.0.22 (MedCalc Software, Mariakerke, Belgium). Categorical variables were compared by the χ2 test. Continuous variables were compared by the Mann‒Whitney U test for abnormally distributed variables and t test for normally distributed variables. The level of significant difference reported was two-tailed, and p values of less than 0.05 were considered statistically significant. The inter/intraclass correlation coefficient (ICC) was used to evaluate interobserver and intraobserver agreement. ICC values were considered excellent for ICC ≥ 0.75, satisfactory for ICC 0.4 ≤ ICC < 0.75, and poor for ICC < 0.4. Z score normalization data processing was performed using R software version 3.4.1 with the caret package, and the glmnet package was used for LASSO regression. The classification learner toolbox in MATLAB R2021b (MathWorks, Natick, MA) was used to build linear SVM models for predicting ETE status. The discrimination metrics of established models, including AUC, classification accuracy, sensitivity, specificity and F1 scores, were calculated using MedCalc.

## Results

### Clinicopathological characteristics

**Fig. 3:**
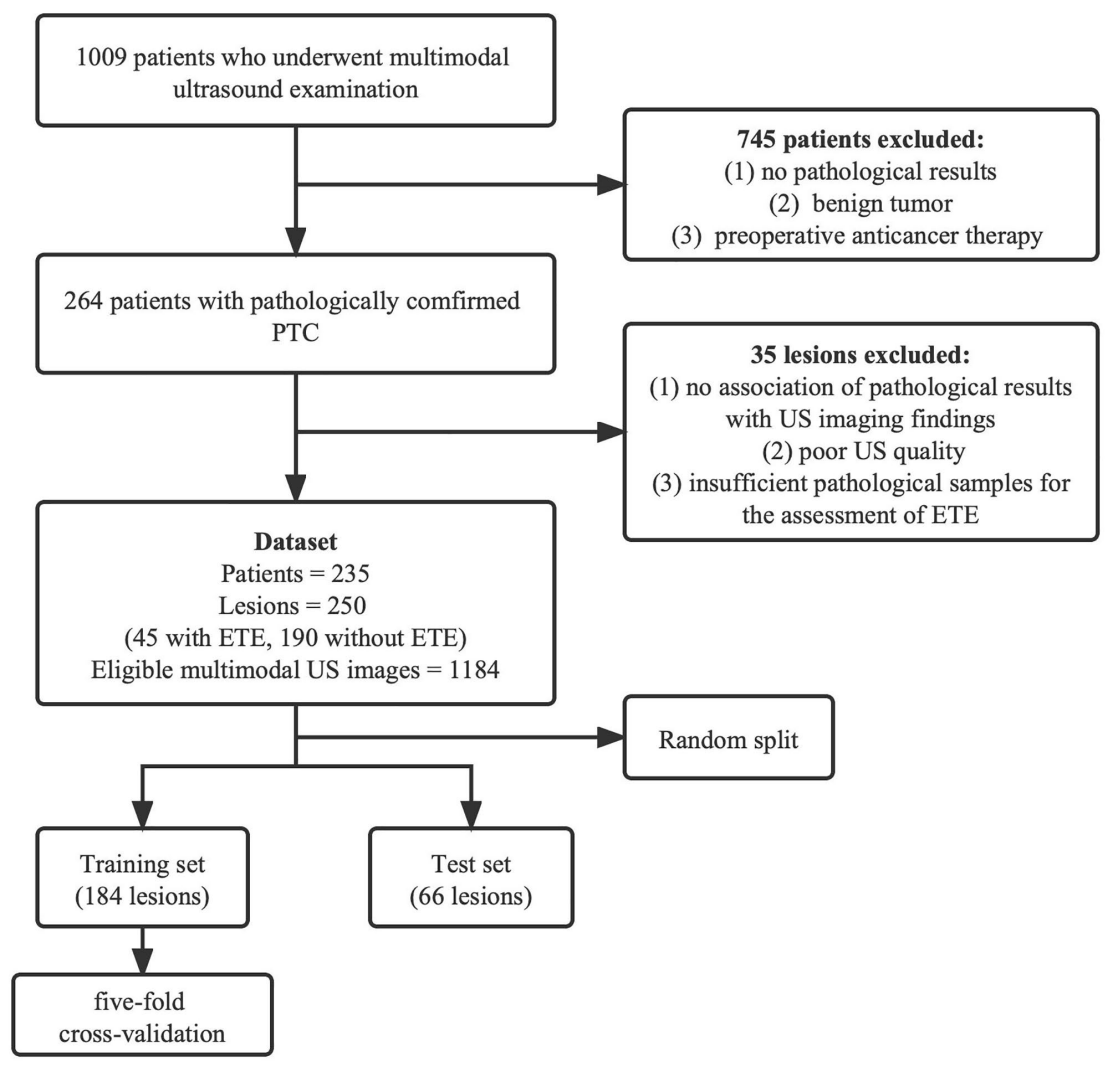
Study flowchart US: ultrasound; PTC: papillary thyroid carcinoma; ETE: extrathyroidal extension

Among the 1009 thyroid patients examined by multimodal US, 235 patients with 250 suspicious thyroid lesions on multimodal ultrasonography who underwent surgery between 01 January 2019 and 30 April 2022 were included (shown in Fig. 3). The 250 lesions were randomly allocated across two sets, namely, a training set and a test set, containing 184 lesions (40 males and 144 females; median age 41.0 years) and 66 lesions (15 males and 51 females; median age 41.5 years), respectively. The clinicopathological characteristics of the training and test sets are shown in Table 1. Positive ETE accounted for 17.9% (33/184) and 18.2% (12/66) of cases in the training and test sets, respectively. There was no significant difference between the two cohorts in the presence of ETE (P = 0.964). In addition, there were no significant differences between the two cohorts in other clinicopathological characteristics. These results justified the use of the training and test cohorts.

**Table 1:** Clinicopathological characteristics of PTCs in the training and test sets

Clinicopathological characteristics	Training set (n = 184)	Test set (n = 66)	P Value
Gender			0.868
Male	40 (16.0%)	15 (6.0%)	
Female	144 (57.6%)	51 (20.4%)	
Age (years)	41.0 (33.0, 51.0)	41.5 (32.0, 53.0)	0.939
Maximum Size (cm)	0.8 (0.6, 1.2)	0.9 (0.6, 1.2)	0.915
Location			0.425
Isthmus	7 (2.8%)	4 (1.6%)	
Left lobe	91 (36.4%)	27 (10.8%)	
Right lobe	86 (34.4%)	35 (14.0%)	
US-LNM			0.177
Normal	146 (58.4%)	47 (18.8%)	
Suspicious	38 (15.2%)	19 (7.6%)	
Pathological LNM			0.227
No	94 (37.6%)	28 (11.2%)	
Yes	90 (36.0%)	38 (15.2%)	
ETE			0.964
No	151 (60.4%)	54 (21.6%)	
Yes	33 (13.2%)	12 (4.8%)	

Data are presented as median with interquartile range and number where applicable

PTC: papillary thyroid carcinoma; LNM: cervical lymph node metastasis; US-LNM: cervical lymph node metastasis on ultrasound; ETE: extrathyroidal extension

### Clinical and multimodal US characteristics

The multimodal US characteristics of the study population are presented in Table 2. Univariate analysis was used to identify potential variables associated with ETE. The Tg level, maximum size, US-LNM, degree of contact with the capsule, angle of contact, bulging, capsule echogenic loss on BMUS, SMI vascularity extending beyond the capsule and discontinuous capsular enhancement were associated with ETE in PTCs (P < 0.05).

**Table 2:** Univariate analysis of clinical and multimodal US characteristics for ETE in the training set

Characteristics	Non-ETE (n = 151)	ETE (n = 33)	P Value
Gender			0.075
Male	29 (15.8%)	11 (6.0%)	
Female	122 (66.3%)	22 (12.0%)	
Age (years)	41.0 (33.0, 51.0)	44.0 (36.0, 52.5)	0.514
Tg			**0.030**
Low	34 (18.5%)	3 (1.6%)	
Normal	112 (60.9%)	26 (14.1%)	
High	5 (2.7%)	4 (2.2%)	
TgAb			0.640
Normal	118 (64.1%)	27 (14.7%)	
Abnormal	33 (17.9%)	6 (3.3%)	
TPOAb			0.206
Normal	118 (64.1%)	29 (15.8%)	
Abnormal	33 (17.9%)	4 (2.2%)	
Maximum size (cm)	0.70 (0.60,1.00)	1.20 (0.85,1.55)	**< 0.001**
Location			0.119
Isthmus	4 (2.2%)	3 (1.6%)	
Left Lobe	73 (39.7%)	18 (9.8%)	
Right Lobe	74 (40.2%)	12 (6.5%)	
US-LNM			**0.047**
No	124 (67.4%)	22 (12.0%)	
Yes	27 (14.7%)	11 (6.0%)	
Degree of contact			**< 0.001**
< 25%	93 (50.5%)	7 (3.8%)	
25-50%	46 (25.0%)	22 (12.0%)	
>50%	12 (6.5%)	4 (2.2%)	
Angle of contact			**< 0.001**
None	70 (38.0%)	3 (1.6%)	
Acute	63 (34.2%)	26 (14.1%)	
Straight or obtuse	18 (9.8%)	4 (2.2%)	
Bulging			**0.014**
No	118 (64.1%)	19 (10.3%)	
Yes	33 (17.9%)	14 (7.6%)	
Capsule echogenic loss			**< 0.001**
No	125 (67.9%)	16 (8.7%)	
Yes	26 (14.1%)	17 (9.2%)	
CDFI vascularity extending			0.419
No	143 (77.7%)	30 (16.3%)	
Yes	8 (4.4%)	3 (1.6%)	
SMI vascularity extending			**0.009**
No	145 (78.8%)	27 (14.7%)	
Yes	6 (3.3%)	6 (3.3%)	
Enhancement extending			0.082
No	145 (78.8%)	29 (15.8%)	
Yes	6 (3.3%)	4 (2.2%)	
Discontinuous capsular enhancement			**< 0.001**
No	127 (69.0%)	18 (9.8%)	
Yes	24 (13.0%)	15 (8.2%)	

Data are presented as median with interquartile range and number where applicable

US, ultrasound; ETE, extrathyroidal extension; Tg, thyroglobulin; TPOAb, thyroid peroxidase antibody; TgAb, thyroglobulin antibody; US, ultrasound; CDFI, colour Doppler flow imaging; SMI, superb microvascular imaging; US-LNM, cervical lymph node metastasis on ultrasound

### ETE prediction

**Fig. 4:**
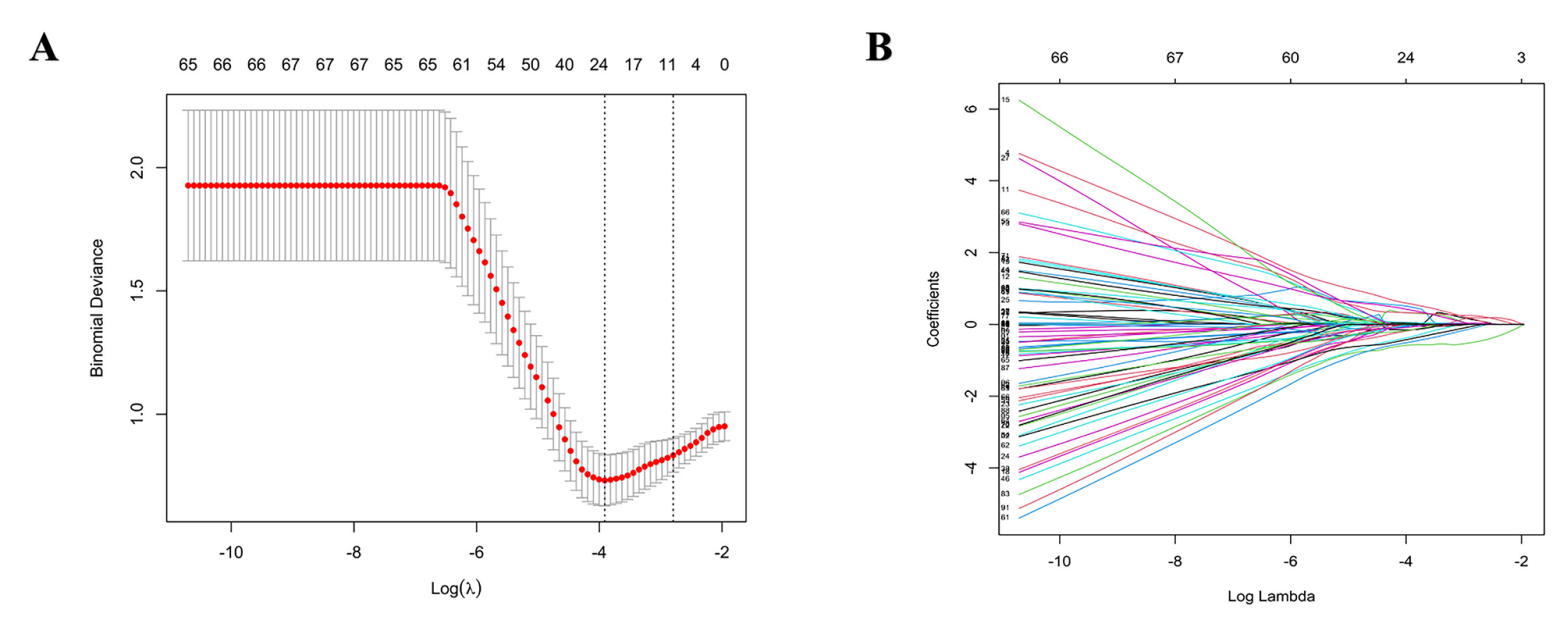
Radiomics feature selection using LASSO regression in the training set (**A**) The 10-fold cross-validation process was used to generate the 13 selected features in LASSO regression for further modelling. (**B**) The feature coefficient convergence graph

Based on the training cohort, a total of 5972 radiomics features were extracted from multimodal sonography. These features were reduced to 180 using F + MI + PA, and LASSO regression was used to select the predictors with the greatest potential (Fig. 4). One shape feature, two BMUS features, three SMI features, three SWE features and four CEUS features were selected to further develop prediction models. The proportion of texture features derived from CEUS was the highest (30.8%). All 13 radiomics features showed highly significant differences between ETE and non-ETE masses with a t test (P < 0.05, Table 3). The radiomic score calculation formula was as follows:

**Table 3:** Comparison of radiomics values of ETE and non-ETE groups in the training set

Modality	Method	Radiomics parameters	Non-ETE (n = 151)	ETE (n = 33)	P Value
BMUS	Geometry	GeoX	(6.26 ± 0.88) × 10^2^	(5.88 ± 1.02) × 10^2^	0.028
	Grey-level co-occurrence matrix	S (5,5) InvDfMom	(1.60 ± 0.30) × 10^− 1^	(1.90 ± 0.40) × 10^− 1^	0.002
	Absolute gradient	GrKurtosis	2.47 ± 1.14	3.20 ± 1.01	0.001
SMI	Grey-level run length matrix	Vertl_GLevNonU	(6.85 ± 5.20) × 10^2^	(12.58 ± 9.07) × 10^2^	0.001
	Wavelets transform	WavEnHH_s5	(7.63 ± 3.18) × 10^1^	(5.78 ± 1.89) × 10^1^	< 0.001
	Wavelets transform	WavEnHL_s6	(1.11 ± 0.6) × 10^2^	(1.36 ± 0.77) × 10^2^	0.036
SWE	Grey-level co-occurrence matrix	H_S (0,1) SumOfSqs	(1.08 ± 0.70) × 10^2^	(1.03 ± 0.88) × 10^2^	0.008
	Autoregressive model	Teta1	(8.8 ± 0.30) × 10^− 1^	(9.0 ± 0.30) × 10^− 1^	< 0.001
	Grey-level run length matrix	R_Horzl_GLevNonU	(5.26 ± 4.21) × 10^2^	(9.64 ± 6.54) × 10^2^	0.001
CEUS	Grey-level run length matrix	Vertl_GLevNonU	(8.14 ± 5.87) × 10^2^	(15.31 ± 12.1) × 10^2^	0.002
	Grey-level run length matrix	Vertl_RLNonUni	(1.98 ± 1.46) × 10^4^	(3.68 ± 2.83) × 10^4^	0.002
	Wavelets transform	WavEnHL_s4	(1.04 ± 0.30) × 10^2^	(0.85 ± 0.20) × 10^2^	< 0.001
	Histogram	Perc.99%	(1.96 ± 0.15) × 10^2^	(1.78 ± 0.25) × 10^2^	< 0.001

BMUS, B-mode US; CDFI, colour Doppler flow imaging; SWE, shear-wave elastography; SMI, superb microvascular imaging; CEUS, contrast-enhanced ultrasound. S(x,y), grey level co-occurrence matrix for inter-pixel distance x along rows and y along columns; InvDfMom, inverse difference moment; Gr Kurtosis, absolute gradient kurtosis; Horzl, Horizontal; Vertl, vertical; GLevNonU, gray-level non-uniformity; WavEnHH (HL), energies of wavelet transform coefficients in frequency channels HH (HL); SumOfSqs, Sum of squares; RLNonUni, run-length non-uniformity; Perc.99%, percentile 99%

Rad-score = -1.842561-0.03930704 × Shape-GeoX + 0.23981317 × BMUS-S (5,5) InvDfMom + 0.29167245 × BMUS-GrKurtosis + 0.08002657 × CEUS-Vertl_GLevNonU + 0.14981715 × CEUS-Vertl_RLNonUni − 0.03253232 × CEUS-WavEnHL_s4-0.56894438 × CEUS-Perc.99% − 0.15750629 × SWE-H_S (0,1) SumOfSqs + 0.03310617 × SWE-Teta1 + 0.26388574 × SWE-R_Horzl_GLevNonU + 0.02669084 × SMI-Vertl_GLevNonU − 0.23046584 × SMI-WavEnHH_s5 + 0.12426557 × SMI-WavEnHL_s6.

The interobserver reproducibility and intraobserver reproducibility of feature extraction are shown in Table 4. All the ICC values were reported to be excellent or satisfactory. Therefore, all the selected radiomics features were input into the radiomics model.

**Table 4:** The inter-observer reproducibility and intra-observer reproducibility of selected feature

Selected features	Interclass coefficient	Intraclass coefficient
Shape-GeoX	0.993	0.986
BMUS-S (5,5) InvDfMom	0.895	0.947
BMUS-GrKurtosis	0.705	0.772
SMI-Vertl_GLevNonU	0.858	0.790
SMI-WavEnHH_s5	0.802	0.862
SMI-WavEnHL_s6	0.668	0.798
SWE-H_S (0,1) SumOfSqs	0.721	0.966
SWE-Teta1	0.402	0.652
SWE-R_Horzl_GLevNonU	0.740	0.774
CEUS-Vertl_GLevNonU	0.816	0.930
CEUS-Vertl_RLNonUni	0.840	0.900
CEUS-WavEnHL_s4	0.858	0.942
CEUS-Perc.99%	0.423	0.908

Inter/intra class correlation coefficient values were considered excellent for ICC ≥ 0.75, satisfactory for ICC 0.4 ≤ ICC < 0.75, poor for ICC < 0.4

**Fig. 5:**
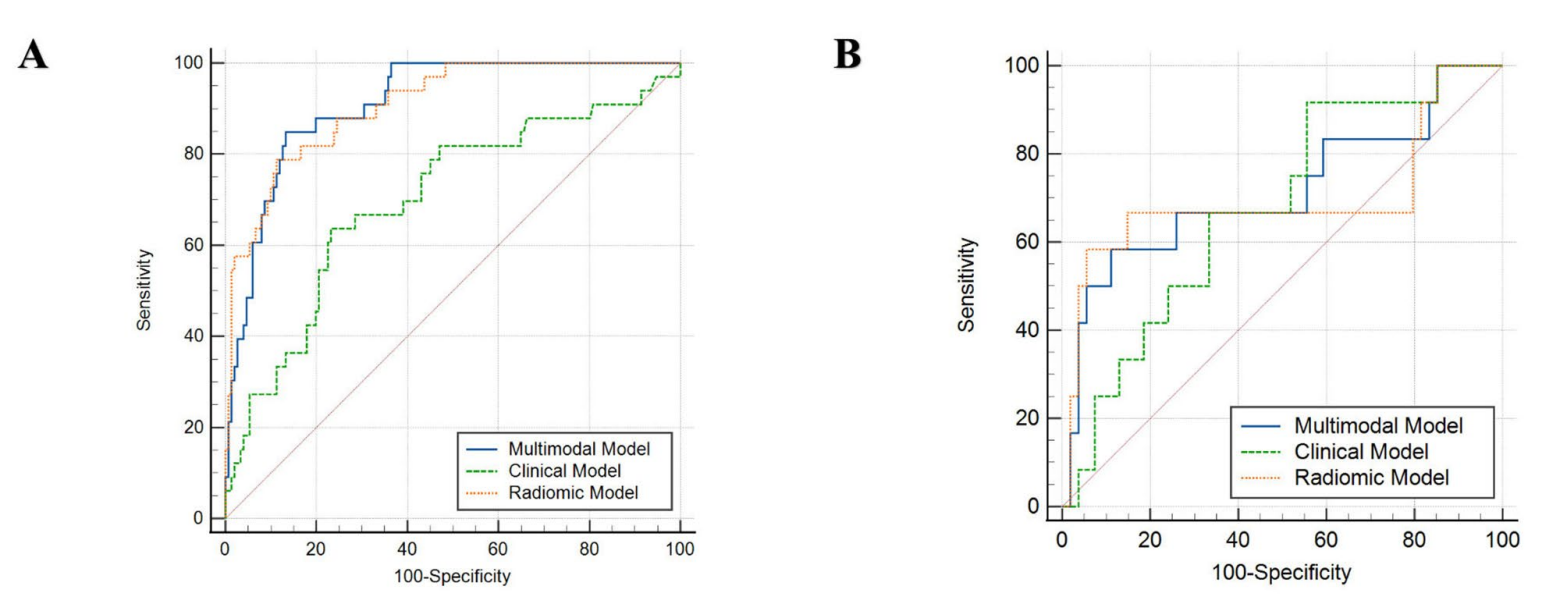
(**A**) ROC curves of the three models in the cross-validation set. (**B**) ROC curves of the three models in the test set

**Fig. 6:**
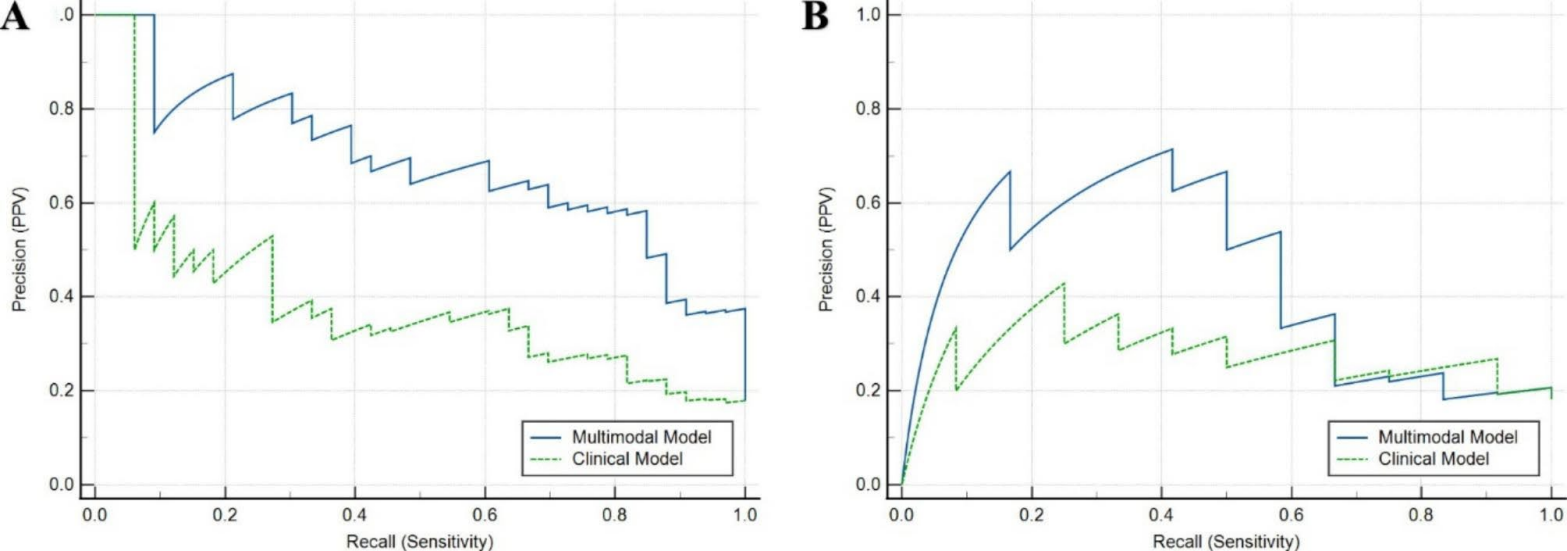
(**A**) The precision-recall curve in the cross-validation set. (**B**) The precision-recall curve in the test set

Figure 5 shows ROC curves for the radiomics model in distinguishing ETE from non-ETE masses in the cross-validation and test cohorts. The multimodal prediction model yielded AUCs of 0.911 (95% CI 0.866–0.957) and 0.716 (95% CI 0.522–0.910) in the cross-validation (Fig. 5A) and test (Fig. 5B) sets, respectively. The clinical decision curve of the multimodal and clinical model is depicted in Fig. 6A and B. Table 5 shows the diagnostic performance of the three models. The sensitivity, specificity, accuracy, AUC and F1 score of the clinical SVM model were 12.1%, 96.7%, 81.5%, 0.700, and 0.472 in the cross-validation set and 8.3%, 96.3%, 80.3%, 0.676, and 0.421 in the test set. The sensitivity, specificity and accuracy, AUC and F1 scores of the radiomics SVM model were 57.6%, 94.7%, 88.0%, 0.908, and 0.691 in the cross-validation set and 58.3%, 94.4%, 87.9%, 0.698, and 0.636 in the test set. Using combined clinical data and radiomics parameters, we developed a multimodal SVM model, which yielded 54.5%, 94.0%, 87.0%, 0.911, and 0.691 in the cross-validation set and 50.0%, 92.6%, 84.8%, 0.716, and 0.571 in the test set, respectively. In the cross-validation cohort, there was a significant difference between the AUC of the multimodal model and the clinical model. However, there was no significant AUC difference among the three models in the test set (shown in Table S1).

**Table 5:** Diagnosis performance of the three models

Group	Model	Sen	Sep	Acc	AUC (95% CI)	F1 score
	Multimodal Model	54.5%	94.0%	87.0%	0.911 (0.866–0.957) *	0.691
Cross-validation	Clinical Model	12.1%	96.7%	81.5%	0.700 (0.593–0.807)	0.472
	Radiomic Model	57.6%	94.7%	88.0%	0.908 (0.857–0.960) *	0.691
	Multimodal Model	50.0%	92.6%	84.8%	0.716 (0.522–0.910)	0.571
Test set	Clinical Model	8.3%	96.3%	80.3%	0.676 (0.513–0.839)	0.421
	Radiomic Model	58.3%	94.4%	87.9%	0.698(0.477–0.918)	0.636

*Compared to AUC of Clinical Model in cross-validation cohort, there is a significant different (P < 0.05). Sen, sensitivity; Spe, specificity; Acc, accuracy; AUC, area under the curve; CI, confidence interval

## Discussion

ETE suggests the aggressive behaviour of thyroid cancer. Previous studies have shown insufficient sensitivity and accuracy in ETE assessment before surgery. Another question is that the US evaluation seems more subjective and relies on expert opinion, which may cause poor interobserver agreement in capsule invasion diagnosis [23]. It is very urgent to utilize modern techniques to improve ETE diagnostic accuracy in preoperative thyroid US.

The present study was designed to determine the effect of multimodal US on ETE prediction before surgery. Researchers have investigated ETE by BMUS in prior studies, defining ETE as contact of more than 25% of the lesion with the thyroid capsule or loss of capsular hyperechogenicity, consistent with our study [24]. It has also been reported that vascularity beyond the capsule on CDFI images showed high specificity but low sensitivity [25]. Conversely, we did not find a relationship between CDFI vascularity and ETE. SMI is a mature method to display tiny blood flow [26]. It reached higher sensitivity in detecting microvessels of thyroid tumours, possibly being more valuable in predicting ETE. In the present study, with a larger sample size, we found that SMI vascularity performed better in diagnosing ETE than CDFI. CEUS has shown a solid ability to distinguish malignant and benign lesions by dynamically exhibiting blood supply patterns. The normal thyroid capsule manifests high enhancement during CEUS because of the vessels in the intrinsic capsule. Like Yan Zhang et al., we found that discontinuous enhancement of the thyroid capsule was highly suggestive of ETE [16]. However, lesion enhancement beyond the capsule could not predict ETE. This may be because few PTCs present hyper or isoenhancement. Moreover, several studies have demonstrated the correlation between LNM and ETE. Our study found that suspicious lymph nodes in cervical US examination also indicate the possibility of ETE.

**Fig. 7:**
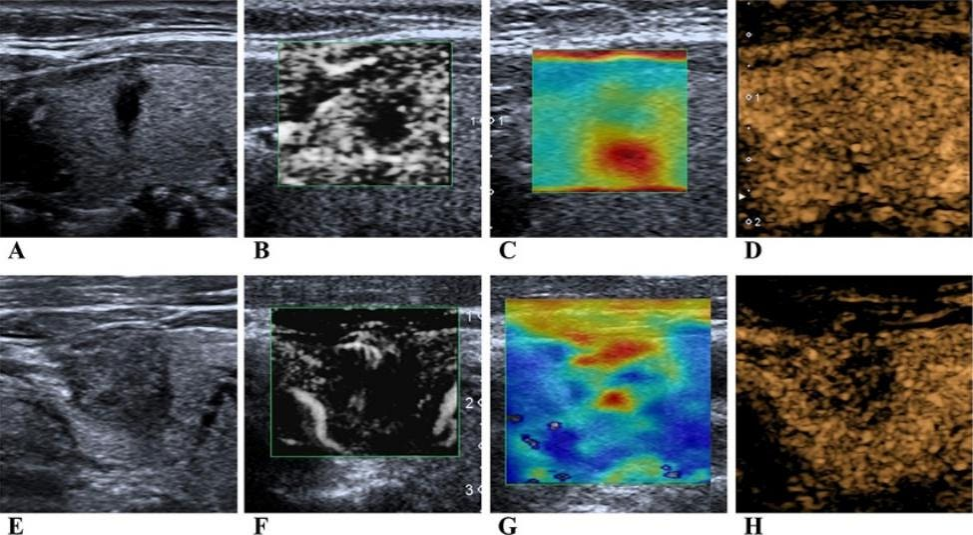
** A** ~ **D**, multimodal ultrasound images for a non-ETE tumour misdiagnosed by the multimodal model but correctly diagnosed by the clinical model; E ~ H, an ETE case misdiagnosed by clinical models but diagnosed correctly by radiomics

Human vision has a limitation in distinguishing details in medical imaging, which causes limited applicability in ETE diagnosis. The clinical model established by linear SVM for ETE prediction showed insufficient discriminatory ability. To overcome this problem, we employed radiomics. Radiomics is known to rapidly extract numerable quantitative features from digital images through high-throughput computing for analysis [27]. It can be widely applied in medical imaging diagnosis and treatment decision-making. The experimental results show that radiomics features extracted from multimodal US images were independently associated with ETE. Adding these radiomics features to the clinical model increased the accuracy, AUC and F1 score in the cross-validation and test sets, which is also better than the previously reported clinical model based on US [7]. Therefore, our study suggested that radiomics has a favourable discriminatory ability to solve medical imaging classification problems. We also investigated cases with inconsistent predictive results from the clinical and multimodal models. Figure 7 provides a series of multimodal US images for two misdiagnosed cases. We speculated that the multimodal model enhances the sensitivity of ETE prediction, which may lead to overestimation or misdiagnosis of minor nodules with a low risk of ETE. On the other hand, the multimodal model still showed better performance than the clinical model, while the thyroidal capsule seemed to be continuous on BMUS imaging (Fig. 7E).

Previous studies showed that the computed tomography (CT) radiomics nomogram for preoperative prediction of ETE in PTC patients was slightly better than that in our research, with an AUC of 0.80–0.84 in the test set [28–30]. Wei R et al. used multimodal magnetic resonance imaging (MRI) radiomics scores to calculate the risk of ETE in PTCs with tumour diameters ≥ 5 mm, which reached an AUC of 0.87 [31]. Given the radiation of CT and renal unsafety of iodine-based contrast media in patients with chronic renal insufficiency and the cost and consuming time of MRI, we prefer to explore a predictive model based on US. However, there needs to be more studies investigating ETE by US imaging radiomics analysis. Although Wang X et al. developed a radiomics nomogram based on the BMUS radiomics score and clinical factors (tumour location and radiologist diagnosis) that reached good discrimination in the validation cohort (AUC = 0.824) [9], an external test set is needed to confirm the generalization ability. In the present study, the SVM-supervised classifier was chosen rather than the nomogram for automatic computational analysis of the possibility of ETE. It performed with high accuracy and a good guarantee against overfitting. The multimodal model based on multimodal US radiomics features and clinical records by linear SVM yielded a cross-validation AUC of 0.911 and a test AUC of 0.716, indicating that multimodal US contained valuable information about ETE. *Table*S2 provides a detailed comparison of the data that have been reported previously for ETE prediction.

However, the number of mETE samples in this study was much larger than that of gETE samples. There were 41 nodules with mETE and 4 with gETE. Thus, this research used the same model to predict both of them. We utilized the subgroup accuracy to assess the predictive ability in different subgroups [subgroup accuracy = the number of correct classifications/total number of samples in the subgroup (%)] (Table 6). The model could predict mETE as well as gETE. Fisher’s exact test showed no difference between the subgroup accuracy of the multimodal model (P value of the cross-validation: 0.489; P value of the test set: 1.00).

**Table 6:** The predictive accuracy of gETE and mETE group in different models

	gETE		mETE	
Models	Cross-validation	Test set	Cross-validation	Test set
Clinical model	0/2 (0%)	1/2 (50%)	4/31 (12.9%)	0/10 (0%)
Radiomic model	2/2 (100%)	2/2 (100%)	17/31 (54.8%)	5/10 (50%)
Multimodal model	2/2 (100%)	1/2 (50%)	16/31 (51.6%)	5/10 (50%)

Number of correct classification/ total number of samples in the subgroup (subgroup accuracy, %)

A major clinical contribution of this paper is providing the possibility of improving individualized treatment in PTC patients. However, the 8th AJCC no longer includes mETE as the protocol to define T3 for tumour staging of PTC. ETE is still a prognostic factor, according to previous studies. Evidence shows that active surveillance is an alternative to surgery in low-risk PTC patients. However, the therapeutic strategies for those with ETE may need to be stricter. The pathological standard for ETE in PTC lesions remains controversial [1, 2]. The incidence of pathological ETE in our study is reasonable due to the prevalence of micro-PTC. Thus, the established model may provide better application in the real world.

Our study has several limitations. First, none of the qualitative multimodal features (e.g., peak intensity, time to peak, SMI vascularity index, and shear wave speed) were included in our study. Further research should use these parameters as input in predictive models. Second, it was a retrospective, one-centre study with unavoidable selection bias. More prospective and multicentre studies are required to explore the possible relationship between multimodal US features and the presence of ETE. Third, radiomics features extracted from static images lead to missing some of the information, especially for CEUS images. Evaluating the thyroid nodule with dynamic real-time US assessment could provide more accurate ETE results. More importantly, a major decrease in AUC in the test set indicated that the model was overfitted. Although we use 5-fold cross-validation to minimize overfitting when modelling, there is still a risk of overfitting in machine learning. This is more likely if the sample size is small and unbalanced. Thus, if sufficient training examples are provided, a deep learning system capable of video image processing will be able to address this issue in the future.

## Conclusions

The diagnostic power of the clinical model in ETE prediction was inferior to the diagnostic power of the radiomics model based on multimodal US. The superiority of multimodal US radiomics features in predicting ETE provides further evidence of the potential clinical value of radiomics analyses in assessing ETE. The linear SVM predictive model based on information supplied by multimodal US radiomics and clinical features demonstrates a promising approach for predicting preoperative ETE in PTC patients.

## Electronic supplementary material

Below is the link to the electronic supplementary material.

Supplementary Material 1

Supplementary Material 2

## Data Availability

The datasets generated and/or analyzed during the current study are not publicly available due to potential comprise of patient’s privacy but are available from the corresponding author on reasonable request.

## Abbreviations

PTC: Primary papillary thyroid carcinoma
ETE: Extrathyroidal extension
mETE: Micro extrathyroidal extension
gETE: Gross extrathyroidal extension
US: Ultrasound
LNM: Cervical lymph node metastasis
BMUS: B-mode ultrasound
CDFI: Color doppler flow imaging
CEUS: Contrast-enhanced ultrasound
SWE: Shear wave elastography
SMI: Superb microvascular imaging
LASSO: Least absolute shrinkage and selection operator
SVM: Support vector machine
ROC: Receiver operating characteristic
ACC: Accuracy
AUC: Area under the curve
95% CI: 95% confidence interval
