# Supplementary material for: Preoperative prediction of extrathyroidal extension: radiomics signature based on multimodal ultrasound to papillary thyroid carcinoma
Source: BMC Med Imaging. 2023 Jul 20;23:96. doi: 10.1186/s12880-023-01049-8 (PMC10360306; doi:10.1186/s12880-023-01049-8)
Supplement: Supplementary file 1 — Supplementary Material 1 [file 12880_2023_1049_MOESM1_ESM.docx]

**Table S1.** P value for AUC difference among three models.

| Models | Cross-validation | Test set |
| --- | --- | --- |
| Multimodal_Model ~ Clinical_Model | <0.0001 | 0.702 |
| Multimodal_Model ~ Radiomic_Model | 0.820 | 0.591 |
| Clinical_Model ~ Radiomic_Model | <0.0001 | 0.865 |
