# Supplementary material for: Preoperative prediction of extrathyroidal extension: radiomics signature based on multimodal ultrasound to papillary thyroid carcinoma
Source: BMC Med Imaging. 2023 Jul 20;23:96. doi: 10.1186/s12880-023-01049-8 (PMC10360306; doi:10.1186/s12880-023-01049-8)
Supplement: Supplementary file 2 — Supplementary Material 2 [file 12880_2023_1049_MOESM2_ESM.docx]

**Table S2**. Recent studies of predicting ETE status in PTC patients

| **First Author, Year** | **Method** | **Imaging modality** | **Training set** | **Test set** | **Sen (%)** | **Sep (%)** | **Acc (%)** | **AUC** |
| --- | --- | --- | --- | --- | --- | --- | --- | --- |
| Zhang Q, 2014 [13] | Quantitative elastography | BMUS and elastography | PTMCs (n = 138) | - | 78.0 | 60.0 | 70.0 | - |
| Liu Y, 2017 [14] | Quantitative CEUS | CEUS | PTCs with diameter ≥ 5mm (n = 109) | - | 75.4 | 78.9 | 77.1 | 0.817 |
| Chen B, 2019 [30] | Radiomics nomogram | Routine and contrast-enhanced CT | PTCs with diameter ≥ 5mm (n = 437) | Validation (n = 187) | 75.9 | 70.7 | - | 0.812 |
| Hu S,2020 [24] | Radiologist diagnosis | BMUS | PTC nodules (n = 246) | - | 79.7 | 83.3 | 81.9 | - |
|  |  | MRI (T1WI, T2WI, DWI, and CET1) | PTC nodules (n = 246) | - | 76.6 | 93.8 | 86.9 | - |
| Lamartina L, 2021 [7] | BMUS signs | BMUS | Thyroid cancer (n = 378) | - | 70.8 | 81.9 | 78.8 | - |
| Wang X, 2021[9] | Radiomics nomogram | BMUS | PTCs with diameter ≥ 5mm (n = 97) | Internal validation (n = 64) | 64.9 | 74.1 | - | 0.824 |
| Zhang Y, 2021 [16] | CEUS signs | CEUS | PTC nodules (n = 124) | - | 86.5 | 72.2 | 78.2 | 0.794 |
| Wei R, 2021 [31] | Radiomics score | MRI (T1WI, T2WI, DWI, and CET1) | PTCs with diameter ≥ 5mm (n = 92) | Internal test (n = 40) | 75.0 | 80.0 | 78.9 | 0.87 |
| Yu P, 2022 [28] | Radiomics nomogram | Non-contrast CT | PTCs with diameter ≥ 5mm (n = 153) | External test (n = 46) | 69.2 | 90.9 | 84.8 | 0.797 |
| Xu XQ, 2022 [29] | Iodie map–based radiomics nomogram | Dual-energy CT | PTC patients (n = 317) | Internal validation (n = 135) | 74 | 78 | 77 | 0.84 |
| Our model | Multimodal US SVM | BMUS, SMI, SWE, CEUS | PTC nodules (n = 184) | Internal test (n = 66) | 50.0 | 92.6 | 84.8 | 0.716 |

PTMC, papillary thyroid microcarcinoma; TPH, time from peak to one half; T1WI, T1-weighted imaging; T2WI, T2-weighted imaging; DWI, Diffusion weighted imaging; CET1, Contrast-enhanced T1WI. Sen, sensitivity; Spe, specificity; Acc, accuracy; AUC, area under the curve
